# Highly efficient *Agrobacterium rhizogenes*-mediated transformation for functional analysis in woodland strawberry

**DOI:** 10.1186/s13007-023-01078-y

**Published:** 2023-09-23

**Authors:** Huiqing Yan, Dandan Ma, Peipei Yi, Guilian Sun, Xingyan Chen, Yin Yi, Xiaolong Huang

**Affiliations:** 1 School of Life Sciences, Guizhou Normal University, Guiyang, 550001 China; 2 Key Laboratory of Plant Physiology and Development Regulation, Guizhou Normal University, Guiyang, 550001 China; 3 Key Laboratory of State Forestry Administration on Bioaffiliationersity Conservation in Mountainous Karst Area of Southwestern China, Guizhou Normal University, Guiyang, 550001 China

**Keywords:** *Agrobacterium rhizogenes*, Woodland strawberry, Hairy root, Rosaceae

## Abstract

**Background:**

The diploid woodland strawberry (*Fragaria vesca*) is an excellent model plant for investigating economically significant traits and several genetic resources within the Rosaceae family. *Agrobacterium rhizogenes*-mediated hairy root transformation is an alternative for exploring gene functions, especially the genes specifically expressed in roots. However, the hairy root transformation has not been established in strawberry.

**Results:**

Here, we described an efficient and rapid hairy root transgenic system for strawberry using *A. rhizogenes*. Strain of *A. rhizogenes* MSU440 or C58C1 was the most suitable for hairy root transformation. The transformation efficiency was highest when tissues contained hypocotyls as explants. The optimal procedure involves *A. rhizogenes* at an optical density (OD_600_) of 0.7 for 10 min and co-cultivation duration for four days, achieving a transgenic efficiency of up to 71.43%. An auxin responsive promoter *DR5*_*ver2*_ carrying an enhanced green fluorescent protein (eGFP) marker was transformed by *A. rhizogenes* MSU440, thereby generating transgenic hairy roots capable of high *eGFP* expression in root tip and meristem of strawberry where auxin accumulated. Finally, this system was applied for functional analysis using jGCaMP7c, which could sense calcium signals. A significant upsurge in *eGFP* expression in the transgenic hairy roots was displayed after adding calcium chloride. The results suggested that this approach was feasible for studying specific promoters and could be a tool to analyze gene functions in the roots of strawberries.

**Conclusion:**

We established a rapid and efficient hairy root transformation in strawberry by optimizing parameters, which was adequate for promoter analysis and functional characterization of candidate genes in strawberry and other rosaceous plants.

## Background

Rosaceae is an economically important cultivated species in most regions. Genetic improvement of most rosaceous species is impeded by intolerance to inbreeding, cumbersome genome size, and a long-life cycle. The diploid woodland strawberry (*Fragaria vesca*) does not have these limitations [1]. It is a swiftly maturing herbaceous perennial featuring a short reproductive cycle and small plant size [2]. Its small genome sequence has expedited the exploration of gene functions that regulate the growth and development of Rosaceae, offering a model within the Rosaceae family and rendering it possible to identify genes responsible for genetic investigations, especially desirable economic traits [3, 4].

Two fundamental genetic strategies are used to investigate gene functions. A forward genetic method commences with the observation of mutants and then the identification of the responsible genes. The other reverse genetic approach begins with a candidate gene and subsequent transformation of the appropriate genotype [5]. The *Agrobacterium tumefaciens*-mediated genetic transformation system has extensively explored strawberry gene functions. Transgenic strawberry (*Fragaria*×*ananassa* Duch, cv. Chandler) plants were obtained via *A. tumefaciens* using in vitro plantlets and confirmed by GUS assay and PCR analysis [6, 7]. Likewise, an efficient *A. tumefaciens*-mediated transformation of woodland strawberry employing hygromycin or geneticin selection was developed for functional genes with a transgenic cycle requiring about 14 ~ 15 weeks, and the efficiency was approximately only 5% [5, 8, 9]. In general, stable genetic transformation is an efficient tool in a targeted and permanent manner. However, the process is often time-consuming, labor-intensive, and less efficient. In contrast, the transient transformation of strawberry enables gene function studies without the regeneration of transformed cells and has the characteristics of time-saving, simplicity, and high transgenic efficiency, such as virus-induced gene silencing (VIGS), *A. tumefaciens*-mediated transient transformation in fruit and so on [10, 11]. The previously reported transgenic strawberry (*Fragaria×ananassa*) was obtained by VIGS via apple latent spherical virus (ALSV) [11]. Besides, an early study showed that overexpression of *FveMYB10* caused fruit coloration by *A. tumefaciens*-mediated transient transformation in fruit [10].

*Agrobacterium rhizogenes* also called *Rhizobium rhizogenes* is a gram-negative soil-born bacterium that infects various plants [12]. It induces highly branched hairy roots upon wounding [13]. Due to the transfer of hairy root-inducing plasmid containing DNA encoding root locus (*rol*) gene, including loci *rolA*, *rolB*, and *rolC*, the region of root-inducing (*Ri*) between the right and left T-DNA regions is required for the introduction of exogenous genes that can integrate into the plant genome [14]. Hairy root transformation mediated by *A. rhizogenes* has emerged as a feasible alternative to traditional transformation and breeding strategies, owing to its ability to generate transgenic materials [15] rapidly. Hairy root transformation using *A. rhizogenes* is gaining importance for recombinant protein production, secondary metabolites research, and reverse genetics studies in plants [15].

More recently, the regeneration of transgenic plantlets using *A. rhizogenes* has been utilized in diverse plants, such as *Brassica napus* [16], *Crocus sativus* [17], *Glycine max* [18], *Cucumis sativus* [19] and *Gossypium hirsutum* [20]. It is an efficient method for RNAi-mediated knockdown, CRISPR/Cas9-mediated genome editing, protein localization, promoter analysis, and monitoring hormone response [21, 22]. For example, the overexpression of *GmNAC15* in transgenic roots augmented the salinity tolerance of soybeans [23]. Hairy root transformation of *Litchi chinesis* has been applied successfully to assess a key anthocyanin regulatory gene, *LcMYB1*, which increases proanthocyanins, anthocyanins, and flavonols [24]. CRISPR/Cas9 targeting the potato *PHYTOENE DESATURASE* was expressed in hairy roots, and 64%~98% of transgenic hairy roots carried targeted mutations [25]. Moreover, transgenic hairy roots could be nonchimeric as they originate from single cells and can transmit to progeny in potatoes due to stable mutations. The average rate was 38% [25]. Similarly, after seven months of culture, a stable plantlet regeneration of cotton through embryogenesis from transgenic hairy roots [20]. Taken together, *A. rhizogenes*-mediated hairy root transformation would curtail the time and labor inputs required for the transformation system and facilitate analyzing gene functions, especially genes implicated with root growth and development.

In this study, we established a rapid and highly efficient *A. rhizogenes*-mediated hairy root transgenic system for strawberry. The transgenic efficiencies of different types of explants and *A. rhizogenes* strains were assessed, and the transgenic procedure was optimized using an enhanced green fluorescent protein (eGFP) marker. Moreover, the applications of this approach for analyzing specific promoter and gene functions in roots were detected via *DR5*_*ver2*_::*eGFP* and *35S*::*jGCaMP7c*, respectively.

## Materials and methods

### Plant materials and explant preparations

Seedling of Yellow Wonder 5AF7 (YW5AF7) produced from the 7th generation inbred lines of woodland strawberry was used in the study. Mature seeds were surface-sterilized with 75% ethanol for 2 min and washed 5 ~ 6 times with sterile ddH_2_O. After being kept at 4 °C overnight, the seeds were dipped in 2% sodium hypochlorite solution (w/v) with a drop of Tween 20 for 10 min and rinsed 5 ~ 6 times with sterile ddH_2_O. Sterilized seeds were cultured on 1/2 Murashige and Skoog (MS) medium (M404, Phytotech Labs, USA) containing 20 g/L sucrose (Sigma-Aldrich, USA), 3 g/L phytagel (Sigma-Aldrich, USA) and adjusted pH = 5.8 [26] at 4 °C for 30 days to break dormancy and transferred to maintain in a growth chamber (16 h light/8 h dark, 350 µmol/ m^2^/s light intensity, 22 °C, and 70% humidity).

The seven-day or fifteen-day after germination (DAG) seedlings grown in vitro were used as the source of explants for the hairy root transformation system, including cotyledon, hypocotyl, seedling cutoff roots (SC, including cotyledon and hypocotyl) from 7 DAG seedlings, as well as leaf and petiole from 15 DAG seedlings. Cotyledons, hypocotyls, leaves, and petioles were cut into 3 ~ 5 mm segments.

### ***A. rhizogenes*strains and vector construction**

Strains of *A. rhizogenes* Ar1193 [27], K599 [28], C58C1 [29], and MSU440 [25] were used to induce hairy roots of strawberries and compared for the transgenic efficiencies. We used MS suspension-infested hypocotyls as a negative control. We constructed *35S::eGFP*, *DR5*_*ver2*_*::eGFP*, and *35S::jGCaMP7c* to test the transformation efficiency, promoter activity, and gene function, respectively.

To construct *eGFP* reporter binary vector *35S*::*eGFP*, the *cauliflower mosaic virus 35S* (*CaMV 35S*) was amplified from JH19 [30] and cloned into binary vectors pDX2181 [31] at *Pst* I and *Hin*d III sites to drive the *eGFP* expression (forward primer: 5’ CCCAAGCTTAGAGATAGATTTGTAGAGAGAGAC 3’; reverse primer: 5’ AACTGCAGTGAGACTTTTCAACAAAGGGT 3’). It also contained hygromycin phosphotransferase (*HPTII*) as a selection marker that conferred resistance to hygromycin.

The auxin response promoter *DR5*_*ver2*_ was cloned into the binary vector pMDC162 before *eGFP*. This promoter activity investigation vector *DR5*_*ver2*_::*eGFP* was kindly provided by Kang’s lab [32].

The *jGCaMP7c* containing *M13-eGFP-CaM* was amplified from pCMV- *jGCaMP7c* to construct vector *35S::jGCaMP7c* [33, 34]. Then, it was inserted into binary vector pMDC32 at *Spe* I and *Kpn* I sites by ClonExpress II One Step Cloning kit (Vazyme, China). The sequences of *jGCaMP7c* were amplified using primers (forward primer: 5’ TCTAGAGGATCCCCGGGTACCATGGGTTCTCATCATCATCATC 3’; reverse primer: 5’ GGCGGCCGCTCTAGAACTAGTTCACTTCGCTGTCATCATTTG 3’).

The above constructors were individually introduced into *A. rhizogenes* strain by freeze-thaw method [35]. The competent cells and 2 µL vector were mixed, frozen in ice for 5 min, and put into liquid nitrogen for 5 min, followed by incubation at 37 °C for 5 min. A 700 µL YEP liquid medium was added and shaken at 220 rpm for 2 h. After centrifuging at 500 rpm for 3 min, 100 µL Luria Bertani broth (LB, Coolaber, China) was added to the residue. Spread the suspension on appropriate antibiotic selection containing LB Agar plates [21].

### ***A. rhizogenes*-mediated strawberry hairy root transformation**

Each *A. rhizogenes* strain was streaked onto LB agar plates supplemented with 50 µg/mL streptomycin and incubated at 28 °C for two days [10]. A single colony was selected into a 5 mL YEP medium containing 50 µg/mL streptomycin, 50 µg/mL kanamycin, and 50 µg/mL chloramphenicol and incubated at 28 °C with agitation (250 rpm) for 24 h [24]. Subsequently, 1 mL of the starter culture was transferred into 50 mL YEP medium for the grown culture. Bacterial cells were centrifuged at 5000 rpm for 8 min and resuspended on varying optical densities OD_600_ of 0.3, 0.5, 0.7, and 0.9 added in MS liquid medium containing 100 µM acetosyringone (AS). *A. rhizogenes* strain at each OD_600_ was inoculated with at least 20 explants, and three biological replicates were performed.

Various explants were submerged in bacterial solution suspended to a final OD_600_ of 0.7 for varying infection time as follows: 5, 10, 20, and 30 min. After removing excess liquid on filter paper, infected explants were transferred onto filter papers wetted with the 100 µM AS and co-cultivated in darkness at 22 °C for 2, 3, 4, and 5 days, individually. After co-cultivation, the infected explants were washed with sterile water and transferred onto MS medium containing 300 mg/Lcarbenicilin and 300 mg/L timentin to induce hairy roots. Afterward, the explants were sub-cultured every two weeks. Each parameter was treated using 20 explants, and three biological replicates were performed. The numbers of callus-regenerated hairy roots and eGFP-positive roots were finally recorded. We calculated the induction rate of callus (number of explants induced callus/ total number of explants × 100%), regeneration rate of root (number of explants regenerated roots/total number of explants × 100%), and frequency of eGFP-positive root (number of eGFP-positive roots/number of regenerate roots × 100%).

### Fluorescence observation of hairy roots

The visualization of GFP fluorescence in transgenic hairy roots was detected using a LUYOR-3415RG dual florescent protein flashlights with a filter cube LUV-495 A 67 mm, excitation wavelength 440–460 nm and emissions wavelength 500 nm (LUYOR, China). All explants, hairy roots, and seedlings were observed using a stereo microscope Olympus SZX16 (Olympus, Japan) equipped with a digital camera Olympus E-330.

Imaging Laser scanning confocal microscopy was carried out with hairy roots immersed in sterile water using the LSM710 laser scanning microscope (Carl Zeiss, German). Imaging was observed according to the preset eGFP emission spectrum with the following set: 20×objective, 1.5-airy unit pinhole, 500 gain, 488 nm excitation wavelengths, and 514 nm emission wavelength.

### DNA extraction and PCR analysis

DNA from regenerated hairy roots of transgenic and non-transgenic roots of YW (MS suspension-infested hypocotyls as a negative control) was extracted using the cetyltrimethyl ammonium bromide (CTAB) method according to Oosumi et al. [8]. First, hairy roots were collected from each explant and ground in a 1.5 mL centrifuge tube with 50 µL extraction buffer with a disposable grinder. Then, 150 µL extraction buffer supplemented with 2% (w/v) polyvinylpyrrolidone (PVP) was added and mixed. After heated 65 ℃ for 30 min, the samples were centrifuged at 12,000 rpm for 10 min. The DNA solution was extracted twice with equal volumes of chloroform: isoamyl alcohol (24:1). DNA was precipitated with two volumes of ethanol at -20℃ overnight. After washing twice with 75% ethanol, the DNA was dissolved in 20 µL TE buffer containing 10 µg/ml RNase.

The fragment of 720 bp *eGFP* was amplified using the primers (forward primer: 5’ ATGGTGAGCAAGGGCGA 3’; reverse primer 5’ TTACTTTGTACAGCTCGTCCA 3’). Besides, the fragment of 1000 bp *HPT II* was amplified using the primers (forward primer: (5’ CTATTTCTTTGCCCTCGGACGAGTGCTGGGGCGT 3’; reverse primer: 5’ ATGAAAAAGCCTGAACTCACCGCGACGTCTGT 3’). The PCR reaction mixture was as follows: 1 µL (~ 10 ng) plant genomic DNA, 10 µL of 2×Dream Taq PCR master mix (Thermo Fisher Scientific, USA), 1 µL of 10 pM forward and reverse primers, and 7 µL ddH_2_O in a final volume of 20 µL. PCR was carried out using the following cycle conditions: 95 °C for 3 min 1 cycle, 95 °C for 30 s, 60 °C for 30 s, 72 °C for 1 min 35 cycles, and a final extension at 72 °C for 5 min using an Applied Biosystems VeritiPro™ Thermal Cycler (Thermo Fisher Scientific, USA). Amplified products were electrophoresed on 1% (w/v) agarose gel containing 0.5 mg/L Gel-Red (Beyotime, China) and visualized using a GelDOc Go imaging system (Bio-Rad, USA).

### Statistical analysis

The data were represented as mean ± standard deviation and compared by analysis of variance (ANOVA) followed by a comparison of means using Turkey’s test using SPSS program version 20.0 (SPSS Inc., Chicago, USA). Values followed by different letters were significantly different at *P* < 0.01.

## Results

### ***A. rhizogenes *strains C58C1 and MSU440 were the most suitable strains for hairy root transformation in strawberry**

**Fig. 1:**
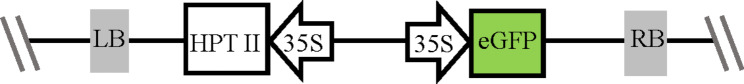
**Schematic representation of** ***35S::eGFP*** **used to test transgenic efficiency**

We used four widely used *A. rhizogenes* strains, including Ar1193, K599, C58C1, and MSU440, to detect which strains were better for hairy root transformation in strawberry. Each *A. rhizogenes* strain harbored a pDX2181 vector that contains eGFP driven by CaMV 35S, and they were used to compare transgenic efficiency (Fig. 1). Hypocotyl of 7 DAG YW was infected with the four strains mentioned above and each harbored *35S*::*eGFP* as a reporter. We used non-transgenic hypocotyls of YW were used as a negative control. In addition, we compared the transgenic efficiencies of different strains using at least 45 explants according to the induction rate of callus formation, regeneration rate of hairy roots, and frequency of eGFP-positive root (Table 1).

**Table 1:** Effects of different types of ***A. rhizogenes*** strains on hairy root transformation of woodland strawberry

**Strains**	**Number of explant cultured** ^**1**^	Number of explants with callus	Number of explants with hairy roots	Number of explants with eGFP-positive root	Induction rate of callus (%)	Regeneration rate of root (%)	Frequency of eGFP- positive root^2^ (%)
Control^3^	45	5.33 ± 1.03	8.67 ± 1.15	0.00 ± 0.00	12.08 ± 2.53^a4^	20.20 ± 6.01^a^	0.00 ± 0.00^a^
Ar1193	53	34.67 ± 2.88	21.00 ± 3.46	0.00 ± 0.00	66.17 ± 10.90^b^	40.20 ± 8.94^b^	0.00 ± 0.00^a^
K599	47	32.67 ± 3.14	23.33 ± 3.21	0.67 ± 0.58	69.68 ± 9.37^b^	49.67 ± 9.37^c^	2.74 ± 2.42^b^
MSU440	48	34.33 ± 7.45	25.33 ± 4.04	11.33 ± 1.15	70.81 ± 7.26^b^	53.36 ± 9.41^c^	45.21 ± 6.27^c^
C58C1	55	37.33 ± 10.48	23.67 ± 6.65	10.67 ± 3.51	66.27 ± 10.16^b^	42.91 ± 9.69^b^	44.68 ± 5.57^c^

1. Infection experiment was repeated three times, hypocotyls from seedling at seven days after germination were used as explants

2. (Number of explants with GFP-positive roots/Number of explants with hairy root) × 100%

3. MS suspension-infested hypocotyls were used as a negative control

4. Means with different letters indicate significant differences (ANOVA test, *p* < 0.01)

*A. rhizogenes* MSU440 came out on top by assessing the induction rate of calli up to 70.81%, followed by K599, C58C1, and Ar1193 (Table 1). Small hairy roots began to emerge at 15 dpi (days post inoculation) and continued to elongate. Then, the number of hairy roots was substantially increased, and the regeneration rate of hairy roots was above 40.20% after inoculation with four *A. rhizogenes* compared to the control. Moreover, we found strong eGFP expression in calli and hairy roots transformed with MSU440 or C58C1, whereas rarely prominent eGFP fluorescence in hairy roots by Ar1193 or K599. The frequencies of eGFP-positive roots (the ratio of the number of hypocotyls with eGFP-positive roots to the number of hairy roots) by MSU440 and C58C1 were significantly higher than the others, which were 45.21% and 44.68%, respectively. By contrast, rarely or only 2.74% of eGFP-positive hairy roots by *A. rhizogenes* Ar1193 or K599, respectively. Thus, C58C1 and MSU440 were the most suitable *A. rhizogenes* strains for hairy root transformation in strawberry.

### **Hypocotyl is the best for *A. rhizogenes*-mediated hairy root transformation in woodland strawberry**

**Fig. 2:**
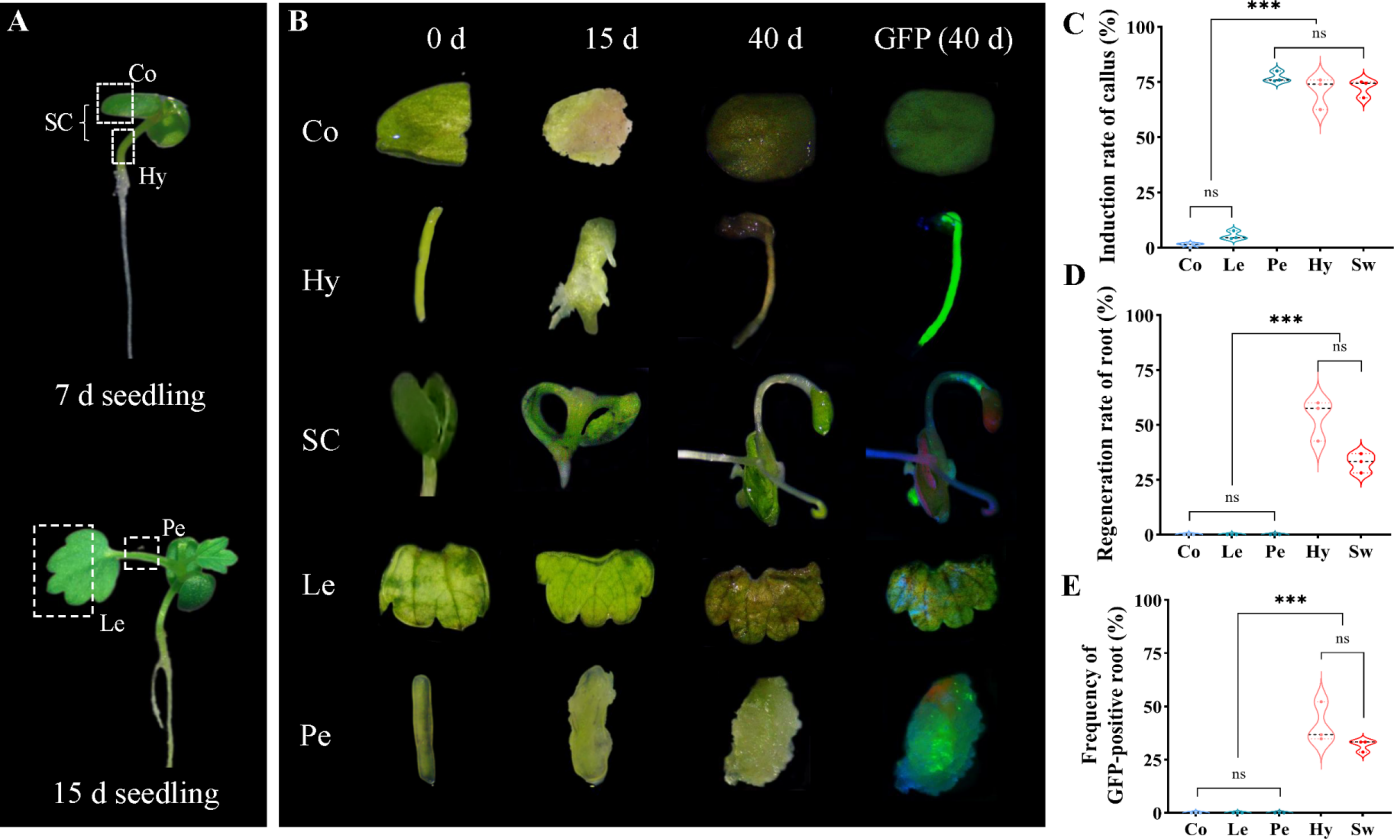
**Efficiency of different explants on hairy root transformation of woodland strawberry. (A)** Five types of explants, including Co, Hy, SC, Le, and Pe, were examined to compare the effects of explant types on the hairy root transformation of woodland strawberry. **(B)** The callus and transgenic roots induction of the five explant types. The matured seeds grow to about 6 cm in height in a 0.7% agar medium. Five kinds of explants were observed on 0, 15, and 40 dpi. eGFP fluorescence on 40 dpi with *A. rhizogenes* strain MSU440 was detected. The induction rates of callus (**C**), root regeneration rate (**D**), and eGFP positive rate (**E**) of five types of explants were compared. ***indicate significant differences (ANOVA followed using Turkey’s test, *P* < 0.01). Co: Cotyledon; Hy: Hypocotyl; SC: Seedling cutoff root, including cotyledon and hypocotyl; Le: Leaf; Pe: Petiole

In order to optimize the protocol of *A. rhizogenes*-mediated transformation, five kinds of explants were used, including cotyledon, hypocotyl, SC (including cotyledon and hypocotyl), leaf pieces, and petioles. The transgenic efficiencies of different explants were compared after *A. rhizogenes* MSU440-mediated hairy root transformation (Fig. 2A).

The ability to induce callus varied based on different kinds of explants. We noticed that calli were rarely induced from cotyledon at 15 dpi, or only small yellow-green nodulars were formed around wound sites of leaf pieces, even at 40 dpi. However, more prominent calli were formed from petioles, hypocotyl, and SC (Fig. 2B). The higher induction rates of these three explants were all above 60%, suggesting their high regeneration ability **(**Fig. 2C). Interestingly, explants involving hypocotyl produced more hairy roots at 15 dpi, and the rate of hairy roots were 53.36% in hypocotyl and 32.77% in SC, whereas rarely regeneration of hairy root in cotyledon, leaf pieces, and petioles (Fig. 2D). The hairy roots underwent elongation. They were used to detect eGFP signals at 40 dpi. A small amount of eGFP fluorescence was observed in transgenic hairy roots from petioles, with the eGFP-positive root frequency of only 4.47%. However, noticeable eGFP fluorescence could be noticed in the hairy roots of hypocotyl and SC. The eGFP-positive rates were up to 41.21% and 31.75%, respectively (Fig. 2E). According to the results, explants involving hypocotyl were the most amenable for hairy root transformation in strawberry.

### **Optimization procedure of an *A. rhizogenes*-mediated woodland strawberry transformation**

To develop a robust hairy root transformation system, we optimized different parameters for hairy root transformation, including *A. rhizogenes* concentration, infection time, and duration of co-cultivation. Hypocotyls were used as explants and infected with *A. rhizogenes* strain MSU440. Then, the callus induction rates, hairy roots rate, and eGFP-positive roots rate were compared.

**Fig. 3:**
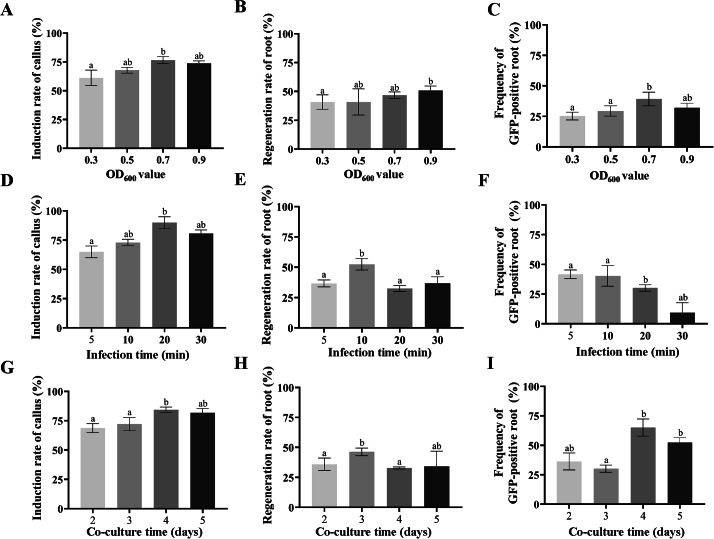
**Optimizing *A. rhizogenes*-mediated hairy root transformation of woodland strawberry.** The rate of callus induction (**A, D**, and **G**), regeneration rate of roots (**B, E**, and **H**), and eGFP positive rate (**C, F**, and **I**) were compared when inoculation with *A. rhizogenes* strain MSU440 of different OD_600_ values, inoculation times, and co-cultivation times, respectively. Different letters indicate significant differences (ANOVA followed using Turkey’s test, *P* < 0.01)

Firstly, gradient concentrations of *A. rhizogenes* of OD_600_ value ranged from 0.3 to 0.9 were compared. The induction rate of callus ranged from 61.14 to 76.67% when the infection time of 10 min and co-cultivation for three days (Fig. 3A). Meanwhile, the hairy root regeneration rate ranged from 40.79 to 50.87% (Fig. 3B). The highest frequency of eGFP-positive hairy roots was 39.26% when OD_600_ was at 0.7 (Fig. 3C).

Afterward, varying infection times from 5 to 30 min were assessed when the *A. rhizogenes* concentration of OD_600_ = 0.7 and co-cultivation for three days. The highest induction rate of callus was 81.41% when infected for 20 min (Fig. 3D). The hairy root regeneration rate of hypocotyls increased significantly from 5 to 10 min, then decreased significantly with the increasing infection times (Fig. 3E). However, the transformation efficiency was the best at 10 min since the frequency of the eGFP-positive root was 40.28% (Fig. 3F).

Finally, different durations of co-cultivation were tested after hypocotyls infected with *A. rhizogenes* when the concentration of *A. rhizogenes* was at OD_600_ of 0.7 and the infected time was for 10 min. The results showed that the callus induction rate after co-cultivation for 4 or 5 days was significantly higher than that of other days, which was 84.41 or 81.77% (Fig. 3G). The regeneration rate of the hairy root at 3 days of co-cultivation was significantly higher than that of 4 or 5 days (Fig. 3H). However, the frequency of the eGFP-positive root was highest, up to 71.43%, when the co-cultivation duration was four days. Hence, four days of co-cultivation duration was the best and was further used for all woodland strawberry transformation experiments. The results suggested that the optimized transformation condition was: *A. rhizogenes* concentration of OD_600_ = 0.7; infection time for 10 min; co-cultivation for four days. Under this procedure, the transgenic efficiency could reach up to 71.43% using hypocotyls by *A. rhizogenes* strain MSU440-mediated hairy root transformation.

### **Phenotype observation and PCR verification of hypocotyls or seedlings cutoff roots infected with *A. rhizogenes *MSU440**

**Fig. 4:**
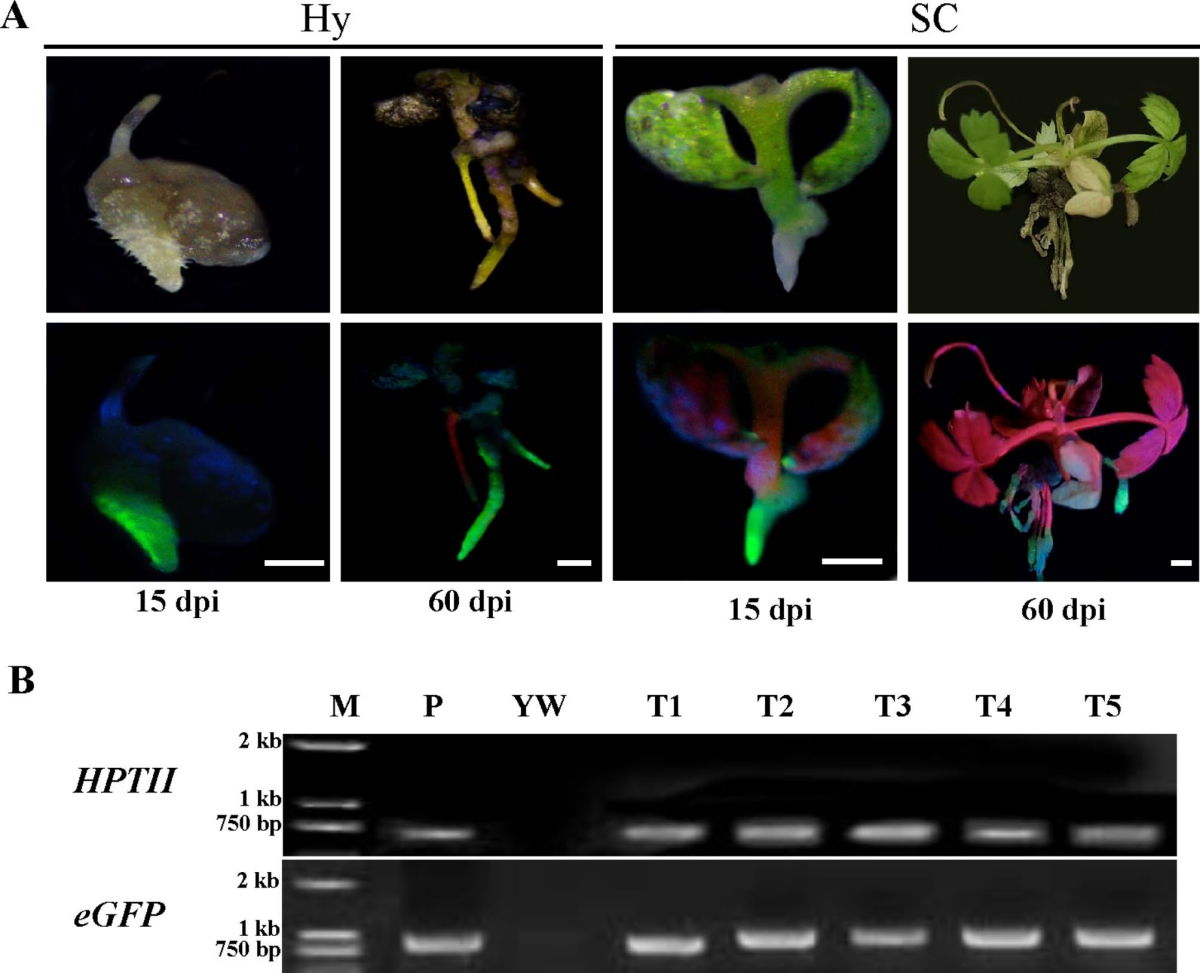
**Phenotype analysis and PCR verification of *A. rhizogenes*mediated hairy root transformation of woodland strawberry. A.** Transgenic roots and corresponding eGFP fluorescence from wounded hypocotyl and SC at 15 dpi and 60 dpi. *(A) rhizogenes* MSU440 loading with *35S*::*eGFP*. Hy: Hypocotyl; SC: Seedling cutoff root (including cotyledon and hypocotyl); Bar = 1 mm. **(B)** PCR analysis for *eGFP* and *HPTII* in five independent transgenic roots. M, 2-kb DNA ladder marker; P: *35S*::*eGFP* plasmid DNA; YW, non-transgenic Yellow wonder DNA (negative control); T1–5, five independent transgenic hairy roots

*A. rhizogenes* strain MSU440 infected hypocotyl or seedlings cutoff roots (SC) from 7 DAG seedlings. Approximate 15 dpi, callus appeared around the wounding site from cutting during explant preparation and about 1 ~ 3 small hairy roots regenerated and continued to elongate (Fig. 4A). Then, the positive transgenic regenerated hairy roots were detected by screening for eGFP fluorescence. Afterward, the apparent signals of eGFP were observed in transgenic hairy roots of hypocotyl or SC at 60 dpi (Fig. 4A).

PCR analysis was also conducted from five randomly selected independent plants to verify the positive transgenic hairy roots. The plasmid of *35S*::*eGFP* was used as a positive control, and non-transgenic roots were used as a negative control. Bands indicating *HPTII* and *eGFP* were not detected in the negative control YW. However, they were detected in the transgenic hairy roots with eGFP fluorescence, indicating that the fragment of the plasmid were successfully integrated into the genome of transgenic hairy roots (Fig. 4B). In summary, *A. rhizogenes*-mediated hairy roots transformation system was amenable for woodland strawberry.

### **The *A. rhizogenes*-mediated hairy root system could serve as a tool to study promoters**

**Fig. 5:**
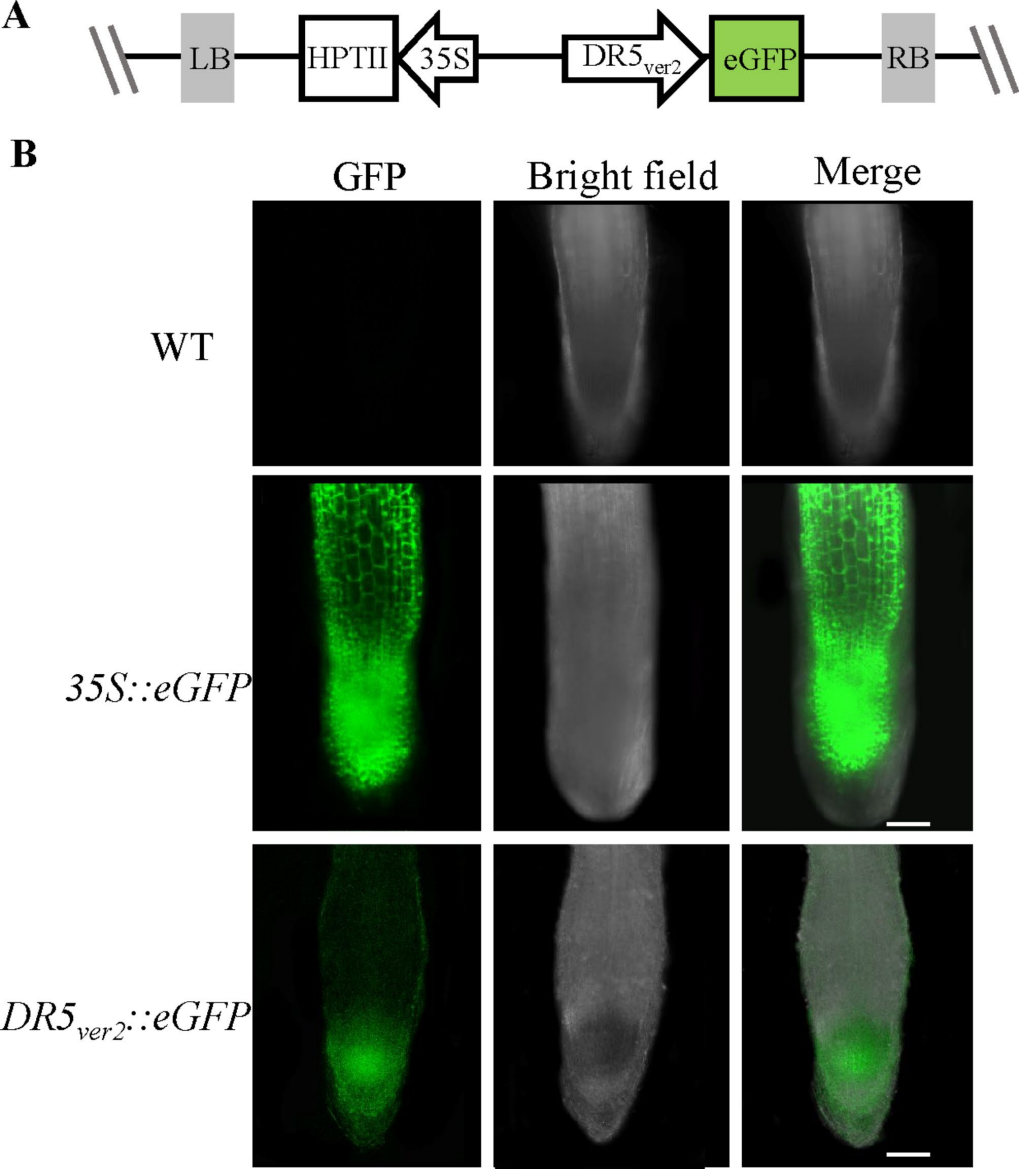
**Promoter activity was analyzed in woodland strawberry hairy roots induced by *A. rhizogenes***. **(A)** Schematic representation of the T-DNA region *DR5*_*ver2*_::*eGFP*. **(B)** Promoter activity is recapitulated in *A. rhizogenes*-transformed hairy roots. No eGFP fluorescence was detected in the transformed hairy root of WT; the 35S promoter drives *eGFP* constituent expression in all the tissue of transformed hairy roots; the *DR5*_*ver2*_ promoter drives strong *eGFP* expression in the root meristem of transformed hairy root. Bars = 100 μm

To explore the application of different promoters mediated by *A. rhizogenes* transformation in strawberry, the constitutive 35S and an auxin-responsive reporter *DR5*_*ver2*_ promoter were used and investigated. *DR5*_*ver2*_ contains nine copies of auxin *cis*-elements, revealing auxin accumulation and signal activity [32]. The intensity of expression is consistent with the auxin distribution since *DR5*_*ver2*_ is an ideal indicator of auxin activity. *DR5*_*ver2*_ was fused to drive *eGFP* expression, and this reporter construct was transformed into YW (Fig. 5A). We used only YW and YW transformed with *35S*::*eGFP* as a negative and a positive control, respectively.

The results showed that strong fluorescence was observed throughout the entire regenerated hairy roots driven by the 35S promoter at 15 dpi, suggesting its constitutive expression in all tissues of hairy roots (Fig. 5B). By contrast, obvious signals of eGFP were only expressed in the tip of regenerated hairy root with a higher level in the root meristem, indicating that *DR5*_*ver2*_::*eGFP* acted correctly in the transgenic strawberry lines due to its expression specifically in the apical meristematic zone of the regenerated root, suggesting its specific expression in the root zone with auxin accumulation. The results indicate that the *DR5*_*ver2*_::*eGFP* reporter behaves appropriately and can be used to monitor auxin distribution in strawberry.

### **Gene function could be assessed by *A. rhizogenes***-**hairy root transformation in strawberry**

**Fig. 6:**
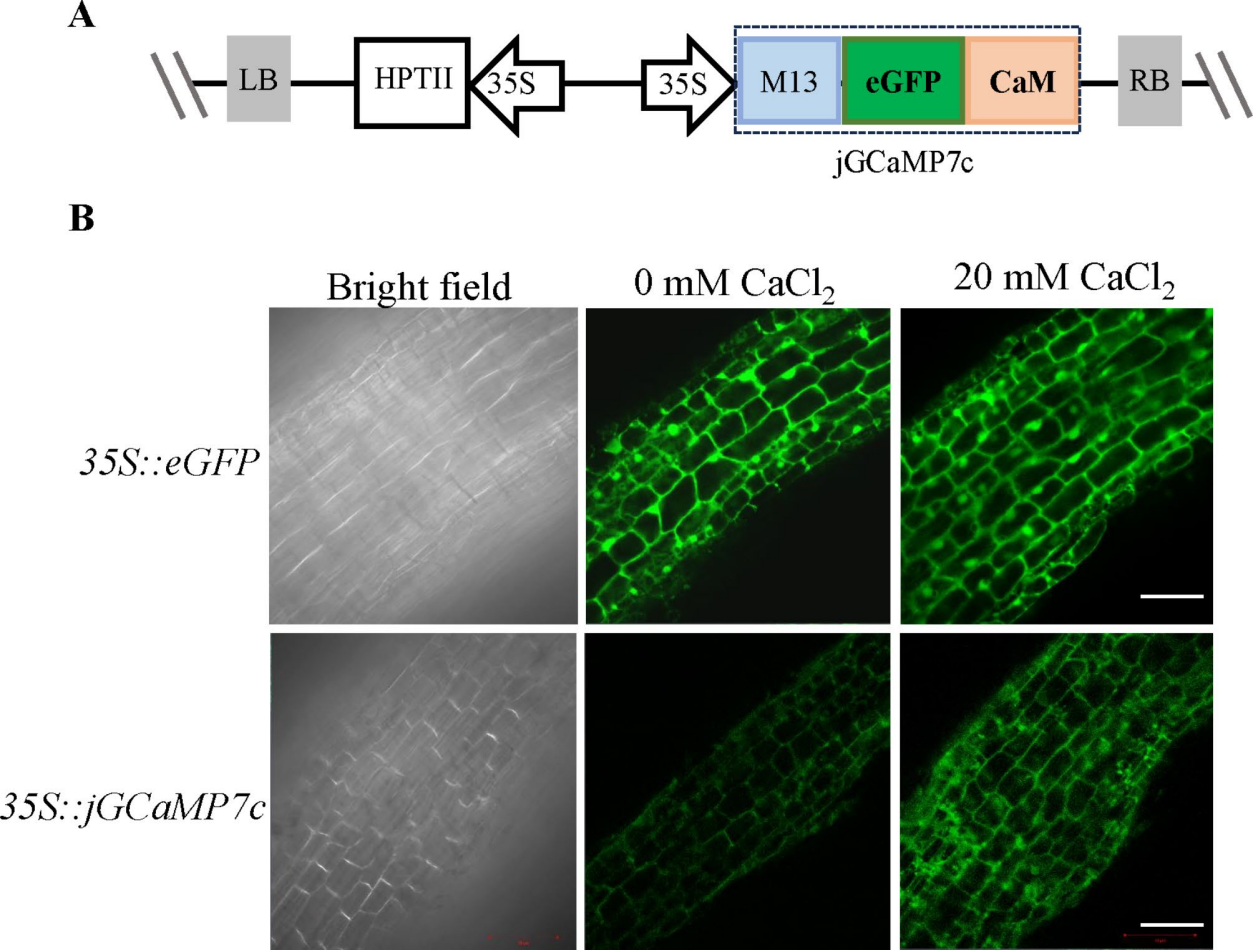
**jGCaMP7c indicated calcium signals in woodland strawberry hairy roots induced by *A. rhizogenes***. **(A)** Schematic representation of *35S::jgCaMP7c*. **(B)** eGFP fluorescence was evenly distributed in the cell of transgenic hairy roots induced by *A. rhizogenes* strain MSU440 loading with *35S*::*eGFP*, and the fluorescence was independent of the existence of calcium chloride (control). The eGFP signal of jGCaMP7c was significantly enhanced in the cytoplasm and cell membrane of transformed hairy roots induced by MSU440 loading with *35S::jgCaMP7c* after adding 20 mM calcium chloride. Bars = 50 μm

In addition to determining specific promoters, we also tested molecular tools for analyzing gene functions in transgenic hairy roots of strawberries. The intensity of the eGFP signal ultimately reflects the alterations in calcium ion concentration by the calcium indicator jGCaMP7c. In addition, the CDS of *jGCaMP7c* were ligated into the overexpression vector pMDC32 and transformed into *A. rhizogenes* MSU440 (Fig. 6A).

Strong fluorescence was detected and distributed in the cells of hairy roots transformed with *A. rhizogenes* MSU440 harboring *35S*::*eGFP*. No apparent alteration was observed in the hairy roots after the addition of exogenous Ca^2+^ (Fig. 6B). By contrast, when transformed by *A. rhizogenes* strain MSU440 loading with *35S::jGCaMP7c*, the detectable eGFP expression was weak and only observed in the cell membrane of hairy roots. Nevertheless, the cytoplasm and cell membrane of transgenic hairy roots expressed an upsurge in eGFP signal after adding 20 mM calcium chloride, indicating that the cytoplasmic Ca^2+^ content was significantly increased. The results confirmed that hairy roots induced by *A. rhizogenes* could be adopted to explore calcium signals in strawberry, thereby providing an efficient way to verify gene functions in the roots of Rosaceae plants.

### **Schematic view of*A. rhizogenes*-mediated transformation of woodland strawberry**

**Fig. 7:**
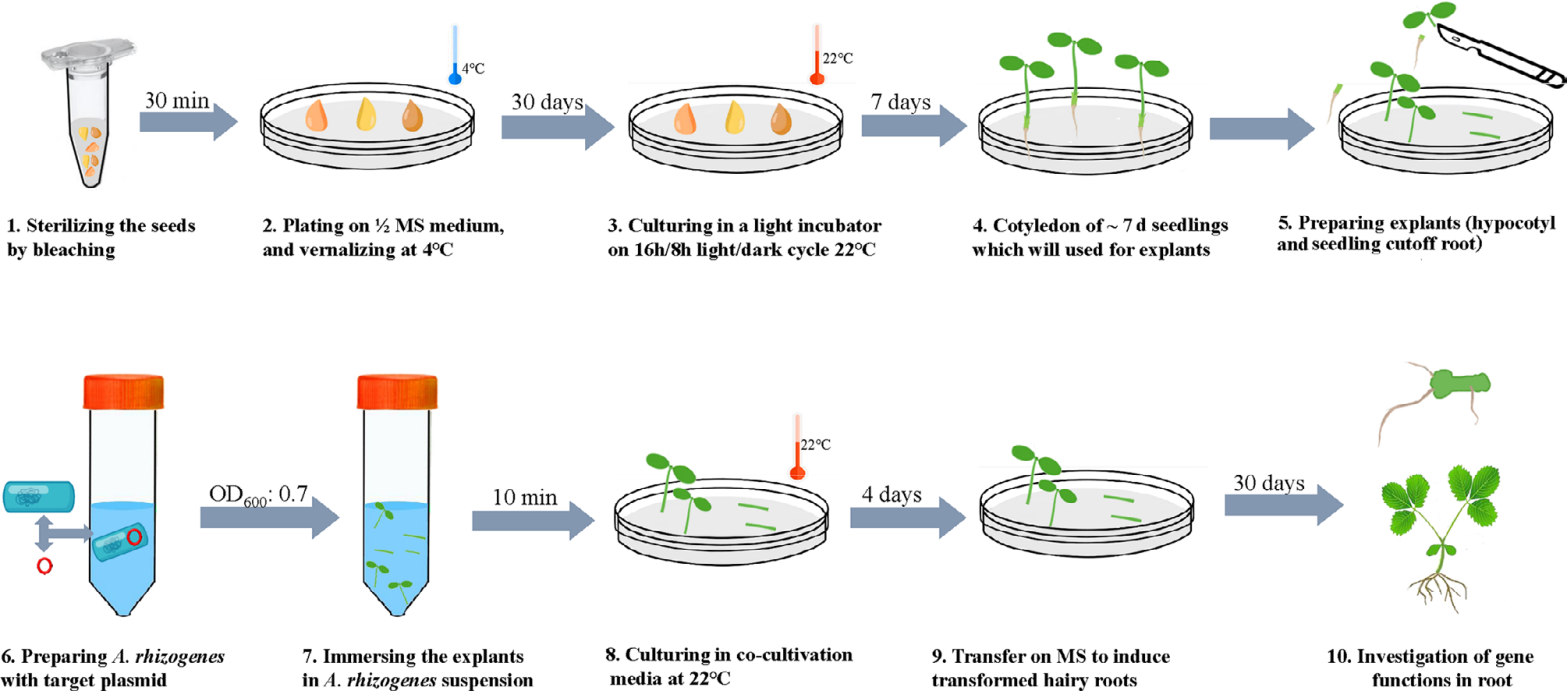
**Schematic view of** ***A. rhizogenes*** **-induced hairy root transformation of woodland strawberry**

A workflow diagram of *A. rhizogenes*-mediated hairy root transformation system in strawberry was displayed in Fig. 7. Briefly, seeds of woodland strawberry were sterilized by bleaching for 30 min and grown on 1/2 MS medium (pH 5.8) at 4℃ for 30 days, then moved to a light incubator on 16 h/ 8 h light/dark at 22℃. Hypocotyls and seedlings cutoff roots excised from 7 d seedlings were used as explants for transformation here. Meanwhile, the binary vectors were transferred into *A. rhizogenes* strain MSU440. Later, wounded tissues were submerged into the suspension of *A. rhizogenes* strain MSU4404 harboring target plasmid at optical density (OD_600_) of 0.7 for 10 min with continuous shaking. Afterward, explants involving hypocotyls were put on filter papers to remove agrobacteria and transferred to the filer papers wetted with MS liquid medium and 100 µM AS, followed by four days duration of co-cultivation in darkness at 22 °C. Finally, the infected tissues were moved to the MS medium to produce transgenic hairy roots.

## Discussion

Strawberry has been promoted as a model due to its unique biological features and amenability to transformation within the Rosaceae family. Fundamental research is imperative to propel transformation frequency in delivering genes and develop a highly efficient and stable regeneration system of strawberry, although great strides have already been achieved [3, 5, 6, 8]. *A. tumefaciens*-mediated genetic transformation of strawberry had been hampered dramatically by their generally poor response to tissue culture and genotype dependence [36]. Time-consuming and low efficiencies have also impeded stable genetic transformation. Compared with *A. tumefaciens*-mediated transformation, *A. rhizogenes* is allowed for rapid functional testing that can be used to determine spatial and temporal aspects of gene expression in root biology [12]. It usually takes less time (approximately 5 ~ 6 weeks) to obtain the hairy transgenic roots, and this system is genotype-independent [20]. A set of candidate genes could be tested using eGFP under the control of a 35S promoter for visual selection of hairy roots of strawberries. The hairy root system would facilitate functional genomics studies within the Rosaceae family.

Factors including types of explants and *A. rhizogenes* strains, *A. rhizogenes* concentration, infection duration, and co-cultivation time influence T-DNA delivery and its integration into the plant genome [24]. The hypocotyl, cotyledon, the shoot apex, petioles, and embryogenic calli could be utilized as materials for transformation [20]. Previous studies have reported that cotyledon as explants produce more hairy roots than hypocotyl segments in cotton and other plants [37]. For example, cotyledon node injection allows faster and more efficient in vivo studies of root development in cucumber than hypocotyl cutting injection [38]. However, in some cases, hypocotyls can yield a higher transformation efficiency that depends on plant species. In this study, the efficiency of explants involving hypocotyl and seedlings cutoff roots (containing cotyledon and hypocotyl) outperformed other tissues of strawberry (Fig. 4). Similarly, an optimal explant for hair root formation in *Toxicodendron radicans* was hypocotyl inoculated with *A. rhizogenes* [39]. Besides, our study showed that MSU440 was the most effective of the four tested strains for the highest eGFP-positive rate.

The *A. rhizogenes*-mediated transformation has also revolutionized to address the localized expression driven by varying promoters in strawberry. For instance, a root cell type- and tissue-specific promoter resource has been generated for *Solanum lycopersicum* and *Solanum pennellii* [40]. *SlCYCD6* and *SlWOX5* promoters show differences in the number of cells marked in the meristem /stem cell niche using hairy root transformation [40]. *DR5*_*ver2*_ reports auxin signaling activity and indirectly indicates auxin levels [41]. Specifically, we used the *DR5*_*ver2*_ promoter to drive *eGFP* expression to reveal auxin distribution in the root of the strawberry. The data exhibited that *DR5*_*ver2*_::*eGFP* was specifically expressed in root tips and responded to auxin application in the root (Fig. 5), indicating that the hairy root transformation is feasible for promoter study.

We used the established hairy roots system to study the function of jGCaMP7c, which detected calcium signals in strawberry. The jGCaMP7c is fused to this calcium probe, and one end of the circularly permuted eGFP is fused with calmodulin (CaM) and expressed at the other end [42]. Fusion expression with myosin light chain kinase M13 (also called RS20) peptide chain that can interact with calmodulin CaM. In this way, when calcium ions bind to calmodulin, it can change the conformation of calmodulin and simultaneously affect the conformation of M13 that interacts with calmodulin. The conformational changes of calmodulin and M13 eventually lead to conformational changes in eGFP, thereby affecting the conformation of eGFP [43]. Furthermore, the overexpression of *jGCaMP7c* in hairy roots demonstrated this versatile and efficient system that will enable the rapid validation of candidate genes for roots of the Rosaceae family.

We successfully established an efficient and rapid hairy root transformation for strawberry. The highest transgenic efficiency was explants involving hypocotyls of strawberry infected with *A. rhizogenes* strain MSU440 at OD_600_ of 0.7 for 10 min and co-cultivation for four days. The hairy root transformation can be a transformative tool to rapidly test promoter activity and gene functions. The hairy root transformation allows genetic improvements to be implemented for the Rosaceae family.

## Data Availability

Not applicable.
